# Supplementary figures and images for: Avidin-biotin complex-based capture coating platform for universal Influenza virus immobilization and characterization
Source: PLoS One. 2021 Feb 26;16(2):e0247429. doi: 10.1371/journal.pone.0247429 (PMC7909696; doi:10.1371/journal.pone.0247429)

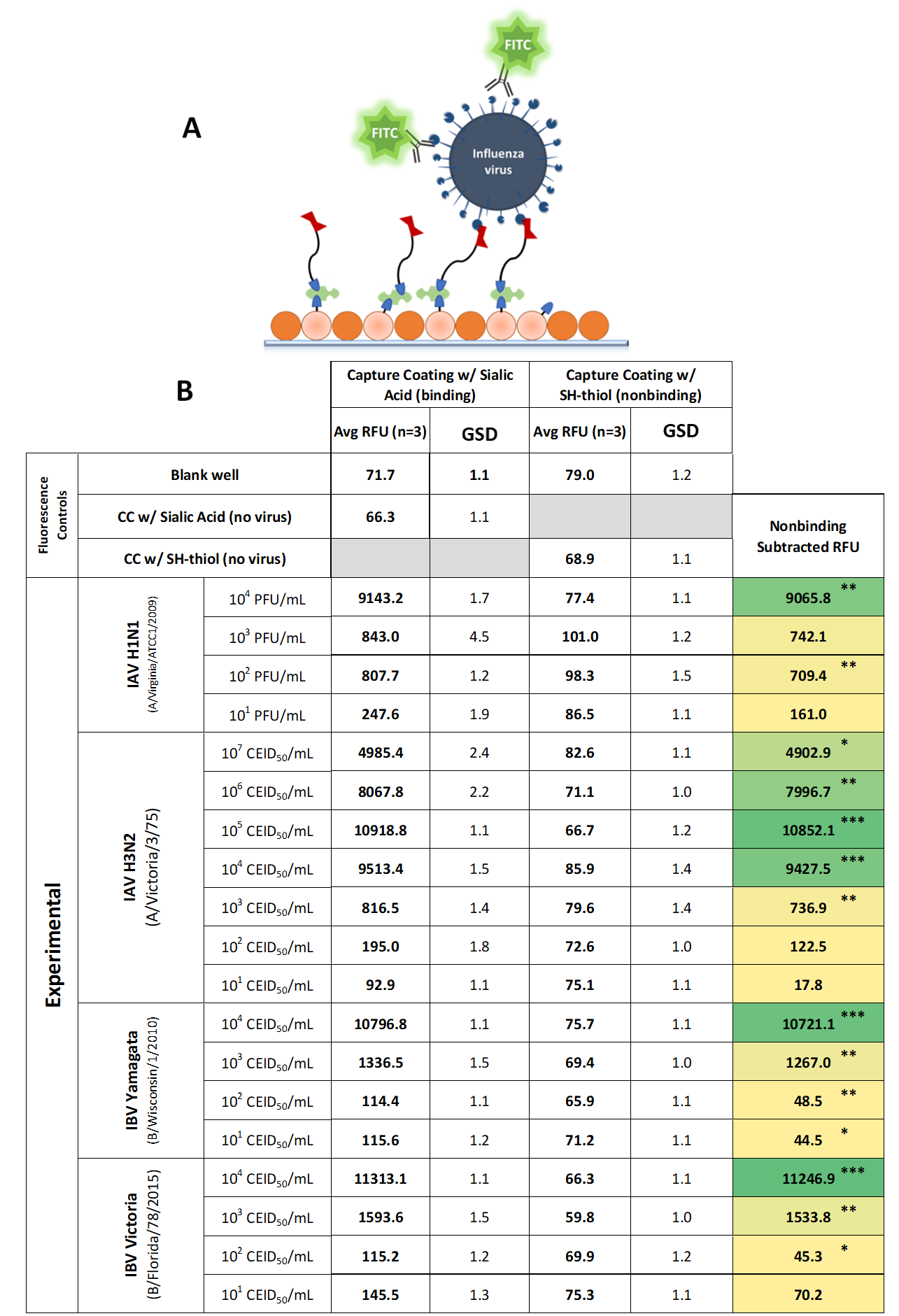

Supplement: S1 Fig — A) Schematic of experimental set up. Influenza virus were incubated on capture coating or control coating, rinsed, and tagged using FITC conjugated anti-Influenza HA antibodies. B) Fluorescence results in relative fluorescence units (RFU). Numbers reported as geometric mean and geometric standard deviation (GSD) (* = p < 0.05, ** = p < 0.01, *** = p < 0.001). (TIF) [file pone.0247429.s001.tif]

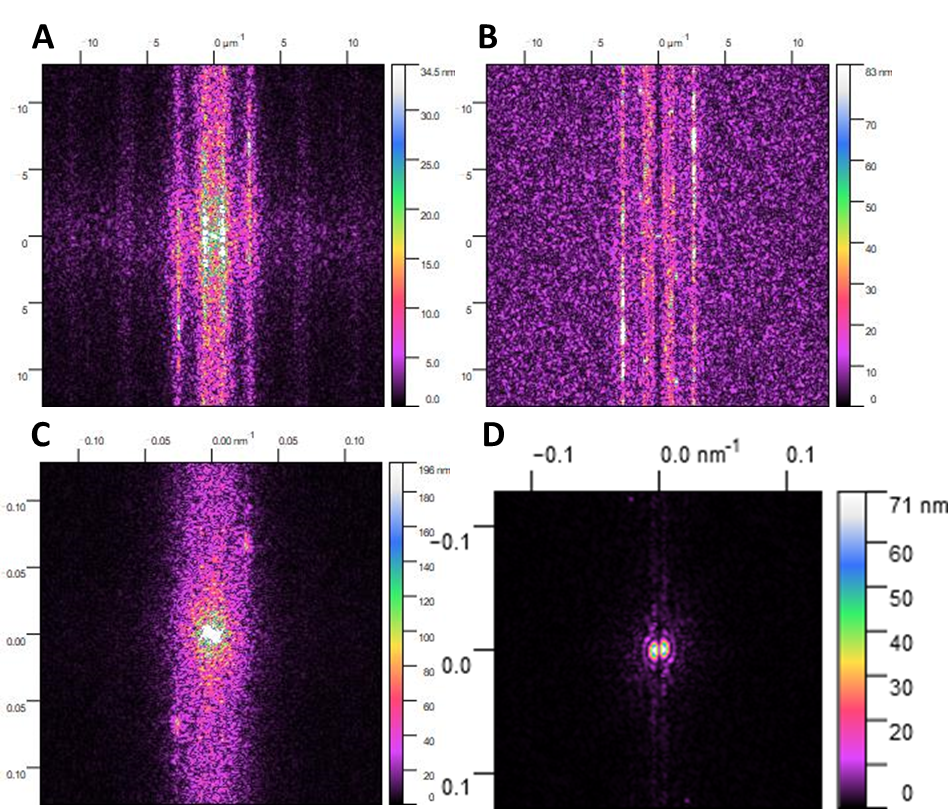

Supplement: S2 Fig — A) 460 μm c-cut sapphire slide. Image suggest spatial features of a repeated, relatively smooth texture. B) Capture coating on sapphire slide. Image suggests spatial features of a repeated, dotted, and quite messy texture. C) Clump of Influenza A H3N2 virion immobilized by capture coting on sapphire slide. The image suggests the virion has a gaussian-like shape with a randomly rough texture within a small variation of the larger gaussian surface. Similarly, D) shows a single Influenza A H3N2 virion immobilized by capture coating on sapphire slide. All 2D FFT analysis was conducted using Gwyddion open source SPM analysis software on captured AFM.tiff images. (TIF) [file pone.0247429.s002.tif]

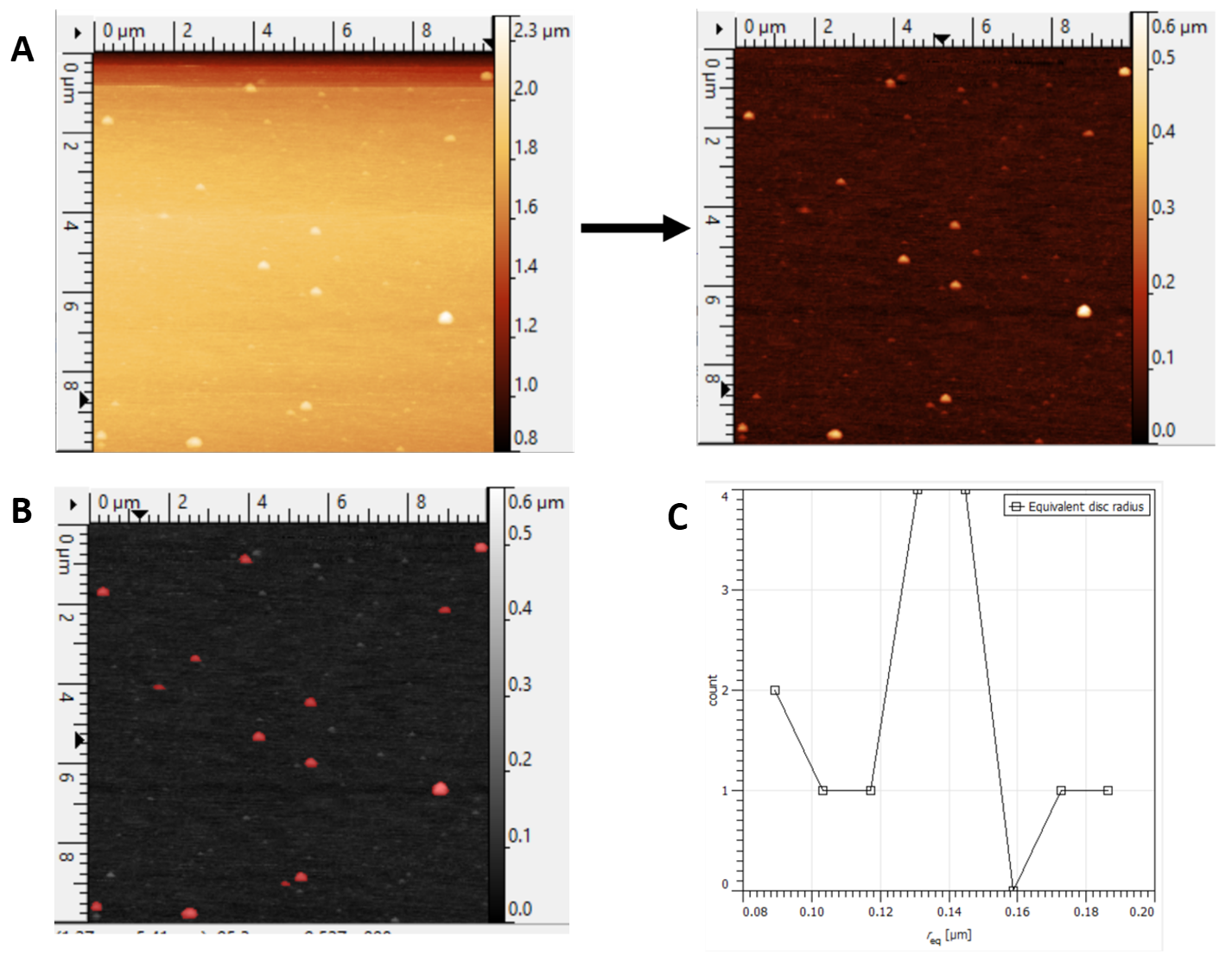

Supplement: S3 Fig — A 10 μm x 10 μm AFM image of immobilized 105 CEID50/mL IBV Yamagata on capture coating was imported processed. (A)The image was leveled using Gwyddion mean plane subtraction, scars corrected, and background subtracted such that the minimum value was set at zero microns. (B) Using the mark grains feature, a threshold was set to 100 nm height and a 70 nm equivalent radius (req) filter applied to account for the 30nm radius AFM probe tip causing broadening edge artefacts of the typically 40–100 nm radius Influenza virus particles. The mask is colored in pink against a grey excluded background. (C) Particles were counted by their req properties. Those with a req larger than 150 nm were double counted as a clustered pair of viruses. This image resulted in a count of 16 IBV Yamagata particles in the given frame. (TIF) [file pone.0247429.s003.tif]

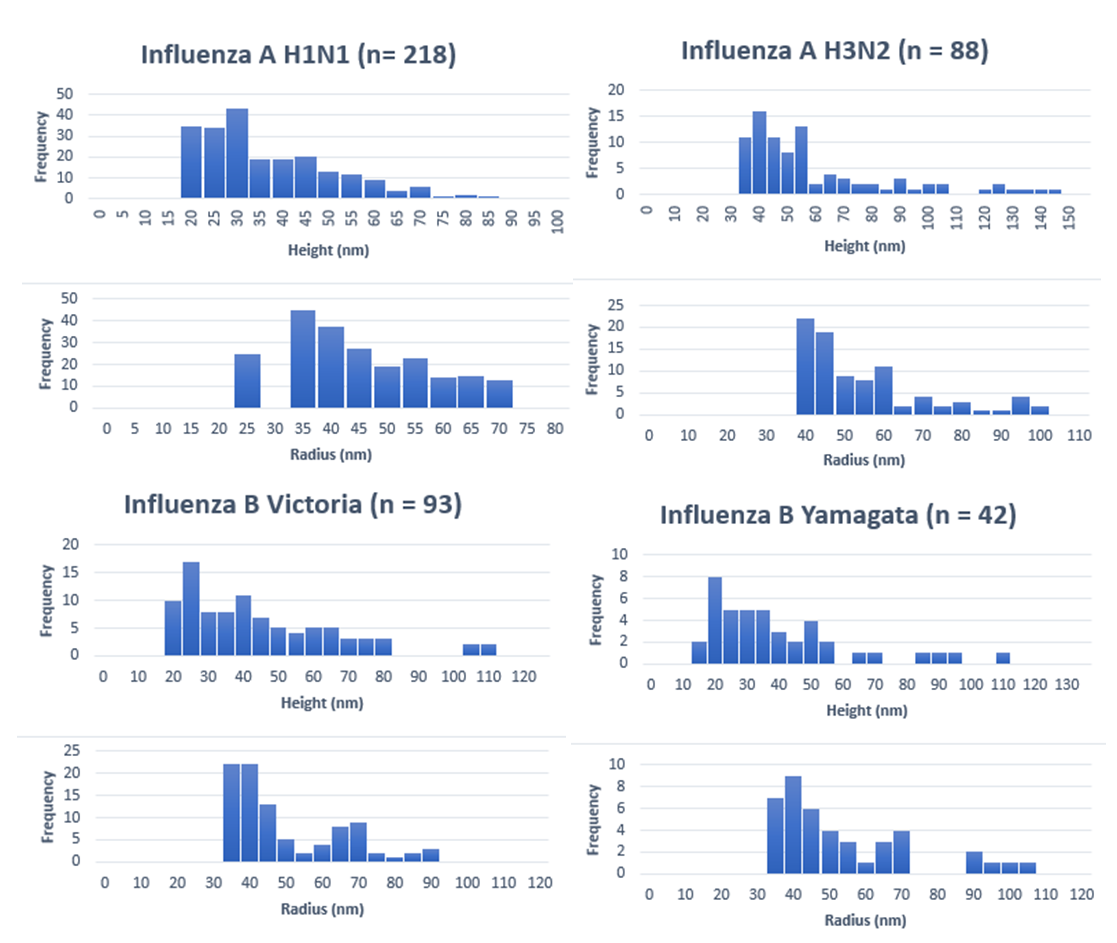

Supplement: S4 Fig — The mean radius and height of each strain was not significantly different than each other strain and were as follows (height, radius): IAV H1N1 (34.16 nm, 42.98 nm), IAV H3N2 (57.75 nm, 53.52 nm), IBV Victoria (41.44 nm, 47.78 nm), and IBV Yamagata (37.68 nm, 51.59 nm). (TIF) [file pone.0247429.s004.tif]

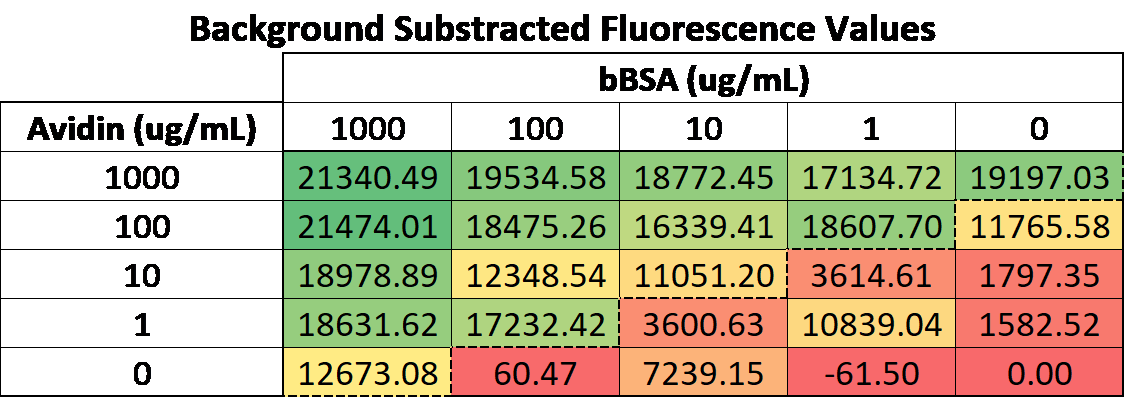

Supplement: S1 Table — Fluorescence values were obtained by subtracting residual fluorescence values from the 0 μg/mL bBSA and 0 μg/mL avidin control and colormetrically scaled. Fluorescent images were captured for each well of a black walled microplate during exposure with an X-CITE 120 fluorescent illuminator fitted with a 480 nm excitation filter with a focal point power of 8.1 mW. Emission was imaged with a SPOT Insight camera through a 40x Nikon Plan Fluor objective and SPOTAdvanced software set to a 519 nm monochrome colorizing palette. Fluorescent unit measurements were made using ImageJ opensource software on the captured images. (TIF) [file pone.0247429.s005.tif]
