# Supplementary material for: Avidin-biotin complex-based capture coating platform for universal Influenza virus immobilization and characterization
Source: PLoS One. 2021 Feb 26;16(2):e0247429. doi: 10.1371/journal.pone.0247429 (PMC7909696; doi:10.1371/journal.pone.0247429)
Supplement: Attachment — Submitted filename: Response to Reviewers.pdf [file pone.0247429.s006.pdf]

WAYNE STATE
School of Medicine & College of Engineering

Manuscript PONE-D-20-36336
Response to Reviewers

January 20, 2021

Etienne Dague, PhD
Academic Editor
PLOS ONE

Dear Dr. Dague and Reviewers,

Thank you for giving us the opportunity to submit a revised draft of the manuscript "Avidin-Biotin Complex Based Capture Coating Platform for Universal Influenza Virus Immobilization and Characterization" for publication in *PLOS ONE*. We are grateful of the time and effort that you and the reviewers have dedicated to providing feedback on our manuscript and are appreciative of the insightful comments which have contributed to valuable improvements to our paper. We have incorporated many of the suggestions made by the reviewers and have supplemented our analysis and discussion with emphasis on originality and AFM evidence for the presence of virus.

Please see below for a point-by-point response to the reviewers' comments and concerns. All line numbers refer to the revised manuscript with tracked changes.

Sincerely,

Micaela Trexler, M.Eng
Doctoral Candidate
Biomedical Engineering
Wayne State University
Detroit, MI 48202

Reviewers’ Comments to the Authors:

Reviewer 1

1. **Comment:** What is the main purpose of this system? For the diagnosis of influenza viruses, there are several methods. Compare the current method with pervious approaches.

   Author Response: Thank you for pointing out the weakness in our claims on originality and for giving us the opportunity to emphasize the novelty and purpose of this work, especially as it compares to current and previous approaches. We have addressed this concern on multiple fronts.

   On the comparison to current diagnostic techniques and how our capture coating may integrate into a novel diagnostic set up, we have added clarifying language and strategic emphasis on the adaptable potential of this work to second half of the Introduction section, **lines 70 to 117.** These paragraphs detail the pros and cons of existing diagnostic techniques (PCR, ELISA, and glycan microarrays) and the progress, potential, and, up to this point, difficulty of molecular spectroscopy techniques that require universal Influenza virus immobilization. Studies using our novel $bPEG_{2k}SA$ capture coating in conjunction with nano-resolution vibrational spectroscopy to differentiate between Influenza virus strains are ongoing, but we felt are outside the scope of this stand-alone manuscript describing the capture coating itself.

   Regarding the novelty of the capture coating itself, we have addressed this in a new paragraph in the Results and Discussion section, **lines 228 to 238.** Here we reference previous studies by Lai et. al (2019), Onkhonova et. al (2019), and Sakai et. al (2017) that used fetuin or mucin as a common method of studying virus binding characteristics and why these endogenous receptors would not be ideal for our future diagnostic purposes:

   > *“Past studies have often used fetuin and/or mucin as a viral receptor, due to an abundance of endogenous sialic acid end chains, in order to study Influenza virus binding characteristics[31-33]. In these cases, the endogenous receptors do not permanently immobilize the virus due to the release mechanism of the vial NA protein. While useful to study binding kinetics, this release mechanism would hinder interrogation by any technique that requires a highly spatially resolved location of the virus, such as super resolution microscopies and spectroscopies. This effect may notably be incompatible in a high laminar flow environment such as a microfluidic. Specifically, in the case of molecular spectroscopies such as Raman spectroscopy and infrared spectroscopy, molecules like fetuin and mucin add an uncontrolled layer of variable molecular makeup that is much more difficult to deconvolve from spectra than well-regulated proteins and molecules like biotin, avidin, and PEG.”*

   In addition, in the following paragraph **lines 239 to 243**, we reference the only previous study (Guo et. al 2018) we are aware of that uses a pegylated sialic aid conjugate for *Influenza virus* immobilization. We use this study as support for the receptor binding functionality of our $bPEG_{2k}SA$ end chain, though our study goals are different.

2. **Comment:** In **Table 1**, why is the fluorescence signal error data so high?

   Author Response: We agree with your assessment that the standard deviation of our fluorescence experiments summarized in **Table 1** is high and have addressed potential reasons with the addition of **lines 206 to 209** in the revised manuscript:

   > *"The high standard deviation of these fluorescence tests, especially at the higher 100 µM bPEG2kSA concentration, may be due to steric hindrance effects caused by a high concentration of receptors. Steric hinderance may be why 10 µM of bPEG2kSA receptor significantly outperformed a higher concentration of 100 µM bPEG2kSA receptor."*

   Reflection on this also motivated us to solidify a claim of significance with new statistical analysis (Welch's t-test, described in Methods section, **lines 152 to 156**), which required handling the statistic calculations with data from the lognormal distribution typical of fluorescence tagged data. This allowed us to claim the optimized $bPEG_{2k}SA$ concentration of 10 µM had "*significantly higher fluorescent reading compared to the 0 µM $bPEG_{2k}SA$ controls (IAV H1N1 $p = 0.041$, IAV H3N2 $p = 0.032$, IBV Yamagata $p < 0.001$).*" (revised manuscript **lines 203-204**) despite the high standard deviation of the data. Values that met the significance criteria are now marked with a "*" in **Table 1** and **S1 Fig**. To reflect these changes, we report the data as geometric mean and standard deviation.

3. **Comment:** For the calculation of capturing efficiency, the related AFM data should be provided.

   Author Response: Thank you for this observation that made us realize our mistake in erroneously omitting the proper standard deviation data in the "Mean Particle Count" column of **Table 3** which is used to calculate the capture efficiency. This data has been added. Further improvements have been made to the AFM section of this manuscript in regard to additional analysis, characterization, and visualization of individual viruses and the capture coating. In the interest of brevity, please refer to our responses to the specific requests of Reviewer 2.

Reviewer 1, thank you again for your invaluable insight and questions pertaining to the originality and data behind this work. We hope we have satisfactorily addressed your concerns on the novelty of our $bPEG_{2k}SA$ and ABC-based capture coating and readily invite additional feedback as you see fit.

Reviewer 2

1. **Comment:** The dimensions of the virus particle in **Fig 2c** does not match established reference work.

   Authors Response: We agree with your assessment, especially as it pertains the topographical profile of the sample in **Fig 2C** that we had erroneously claimed to be a single virion. In response to your insight, we thoroughly reviewed our many AFM images taken over the course of our experiments and determined this image to be a cluster of Influenza viruses, not a single virion. We have added **Fig 2D** to our revised manuscript which provides evidence of a single virion immobilized by the capture coating with a height of 36 nm and a diameter of 160 nm that better

matches the reference studies you provided, given the probable collapsed height due to virus-surface interactions and the edge broadening artefacts of our 30 nm AFM probe. These corrections, with suggested references (de Pablo et. al 2017, Li et. al 2014, and Marchetti et. al 2016), have been added to the revised manuscript, both in a revised **Fig 2** and in **lines 255 to 261**:

> *"Fig 2 displays topographical images and profile analysis results comparing images of the sapphire substrate (Fig 2A), capture coating on sapphire substrate without virus (Fig 2B), and a cluster of Influenza A H3N2 virions immobilized by capture coating on sapphire substrate (Fig 2C), and a single Influenza A H3N2 virions immobilized by capture coating on sapphire substrate (Fig 2D). The measured height of the individual virion, 36 nm, and diameter, 160 nm, agree well with the known size of Influenza virus given the relatively large diameter (~30 nm) of the AFM probe tip causing a broadening edge artefact in the diameter measurement. [37-40] ."*

In addition, we added a new column to **Table 2** with the corresponding characterizing surface parameters for the individual virion in **Fig 2D**. We feel it is important to keep **Fig 2C** as it provides a wonderful visual of the tendril-like structure of the capture coating. However, now it is properly and clearly described as capturing a cluster of viruses as to not mislead the reader.

2. **Comment:** How many individual viruses are like this shown in figure 2?

   Author Response: The vast majority of the virions captured by our coating and imaged using AFM closely resembled the newly referenced profiles in conjunction with **Fig 2D** with heights in the 20 to 60 nm range and radii in the 35 to 70 nm range. We provided evidence for this in response to your next comment below. In all, we were able to evaluate the height and radius dimensions of 218 particles of Influenza A H1N1, 88 of Influenza A H3N2, 93 of Influenza B Victoria lineage, and 42 of Influenza B Yamagata lineage.

3. **Comment:** The authors should provide more statistics about the AFM data, such as size and height distributions. Supplementary **S3 Fig** shows a region with several viruses. How is the statistics of virus heights on the surface?

   Author Response: Prompted by this suggestion, we took advantage of the *mark grain* feature in the Gwyddion software, as used in the capture coating efficiency related particle counts detailed in **S3 Fig**, and applied the same applicable thresholds to all AFM images captured over the course of our various experiments. This included all the 10 x 10 µm scans used in the C_{eff} calculations (including the one shown in **S3 Fig**), gross 50 x 50 µm scans, and detailed 1 x 1 µm scans. Radius and height for each marked grain (i.e. virus particle) were gathered using Gwyddion's *distributions of various grain characteristics* feature and summarized in the histogram plots of the new **S4 Fig** for each of the four Influenza stains used throughout our experiments.

Reviewer 2, thank you again for your insight and for challenging us to look closer at our AFM data. We believe these changes have improved our manuscript greatly and hope we have addressed your concerns aptly. We gladly invite additional feedback as needed.
